# Genomic differences between cultivated soybean, *G. max *and its wild relative *G. soja*

**DOI:** 10.1186/1471-2164-14-S1-S5

**Published:** 2013-01-21

**Authors:** Trupti Joshi, Babu Valliyodan, Jeng-Hung Wu, Suk-Ha Lee, Dong Xu, Henry T Nguyen

**Affiliations:** 1 Department of Computer Science, University of Missouri, Columbia, MO 65211, USA; 2 Christopher S. Bond Life Sciences Center, University of Missouri, Columbia, MO 65211, USA; 3 National Center for Soybean Biotechnology, University of Missouri, Columbia, MO 65211, USA; 4 Informatics Institute, University of Missouri, Columbia, MO 65211, USA; 5 Division of Plant Sciences, University of Missouri, Columbia, MO 65211, USA; 6 Department of Medicine, National Yang-Ming University, Taipei, Taiwan, R.O.C; 7 Department of Plant Science and Research Institute for Agriculture and Life Sciences, Seoul National University, Seoul 151-921, Korea; 8 Plant Genomics and Breeding Institute, Seoul National University, Seoul 151-921, Korea

## Abstract

**Background:**

*Glycine max *is an economically important crop and many different varieties of soybean exist around the world. The first draft sequences and gene models of *G. max *(domesticated soybean) as well as *G. soja *(wild soybean), both became available in 2010. This opened the door for comprehensive comparative genomics studies between the two varieties.

**Results:**

We have further analysed the sequences and identified the 425 genes that are unique to *G. max *and unavailable in *G. soja*. We further studied the genes with significant number of non-synonymous SNPs in their upstream regions. 12 genes involved in seed development, 3 in oil and 6 in protein concentration are unique to *G. max*. A significant number of unique genes are seen to overlap with the QTL regions of the three traits including seed, oil and protein. We have also developed a graphical chromosome visualizer as part of the Soybean Knowledge Base (SoyKB) tools for molecular breeding, which was used in the analysis and visualization of overlapping QTL regions for multiple traits with the deletions and SNPs in *G. soja*.

**Conclusions:**

The comparisons between genome sequences of *G. max *and *G. soja *show significant differences between the genomic compositions of the two. The differences also highlight the phenotypic differences between the two in terms of seed development, oil and protein traits. These significant results have been integrated into the SoyKB resource and are publicly available for users to browse at http://soykb.org/GSoja.

## Introduction

Soybean [*Glycine max *(L.) Merr.] is a chief source of protein and oil for human and animal consumption. It has also become a major source of oil for biodiesel production, as well as a general model to study oil production in plants. The closest variety of soybean to *G. max *is the wild, undomesticated ancestor *G. soja*. The genome of *Glycine soja *has been recently sequenced [1] using Illumina Genome Analyzer and has been shown to have 915.4 MB consensus sequences, covering 97.65% of *G. max *genome sequence [2]. Even though *G. soja *is considered as the closest relative of the cultivated soybean, *G. max*, it has significant phenotypic differences. The large phenotypic diversity in soybean is genetically controlled, both qualitative and quantitative aspects. For example, *G. soja *has mainly tiny, black seeds in contrast to the large yellow seeds of *G. max*. Some QTL differences between *G. max *and *G. soja *have been reported [3-5]. Also, there are very significant differences in the seed oil and protein concentration between wild and domesticated soybeans. Our motivation behind this study is to identify the specific genomic differences that explain these phenotypic changes for the traits of our interest. In *G. soja*, the seed oil concentration is around 8% when compared to the 25% in *G. max*.

Several studies suggest that *G. soja *has important phenotypic characters and specific alleles which are not present in *G. max *[6]. Major traits of agricultural importance including yield and stress tolerance are polygenic and the presence of these favourable alleles in *G. soja *will help breeding program to introduce beneficial traits into soybean [7,8]. Earlier protein QTL studies are the best examples to show that *G. soja *alleles are unique and not present in the *G. max *lines. We have studied the differences between them with respect to things unique to each type as well as differences which make them different from the oil and protein traits perspective. We have also developed graphical tools to simplify the analysis of QTLs and traits, and overlay insertion and deletion regions of one over the other to highlight the genomic differences. This graphical chromosome visualizer is part of Breeding Program tools in SoyKB [9] web resources and provides extensive analysis and visualization capacity with genomics variation data such as SNPs, GWAS, insertions and deletions being overlaid on different QTL regions from multiple traits.

## Materials and methods

### Differences between *G. max *and *G. soja*

We acquired the sequences for all the 20 chromosomes and the gene model predictions for *G. soja *[1] as well as *G. max *[2]. We also acquired the list of inversions and deletions for *G.soja*. The chromosomal start and end positions for each of the 5794 deletions and 194 inversion positions were mapped against the *G. max *reference genome to identify the positions differing between the two. We acquired the sequences of 22,927 contigs assembled from sequences novel to *G. soja*. We also acquired the list of SNPs identified from the MAQ [10] analysis of the sequencing reads for further studies.

### Unique to *G. max*

The 5795 deletion positions were compared against *Glycine max *gene positions for regions of overlap. The genes were considered to be absent in *G. soja *but present in *G. max *if the deleted position of sequences in *Glycine soja *overlapped with more than 50% of the overlapping annotated gene in *Glycine max *using Blast [11]. With this filtering criteria applied, we have identified 425 unique genes from the list of higher confidence ~46K gene model predictions from Phytozome [12] that are absent in *G. soja *and unique in *G. max*. Additional file 1 shows the entire list of these genes identified to be unique to *G. max*. When compared against the entire ~75k gene model predictions the number of genes unique to *G. max *is 2,533.

The 425 genes unique to *G. max *were further analysed to look for overlap with transcription factor predictions from SoyDB [13], functional annotations and the different pathways available in SoyKB [9]. Out of the 425 unique genes, 313 have Pfam domain annotations and 132 can be mapped to various pathways. We identified 12 transcription factors (TFs) amongst this list of unique genes that are present in *Glycine max*, but absent in *Glycine soja *(as shown in Table 1). More than 50% of these TFs belong to the family of C2H2 zinc finger protein, one of the biggest family proteins in the eukaryotes. Members of this family show direct role in plant developmental processes including the embryo development [14] in plants. Also, it has been shown that the TF members from Myb and bHLH families are involved in the secondary axis of branching such as vegetative branches, inflorescence branches, or flowers [15].

**Table 1:** List of transcription factors unique to *G. max*.

Transcription factor	Subfamily
Glyma14g10940	C2H2 (Zn)
Glyma13g37110	C3H-type1(Zn)
Glyma02g03270	CCHC (Zn)
Glyma02g36320	CCHC (Zn)
Glyma04g32310	CCHC (Zn)
Glyma07g07940	CCHC (Zn)
Glyma15g42470	CCHC (Zn)
Glyma18g33480	CCHC (Zn)
Glyma10g10640	MADS
Glyma08g45260	MYB/HD-like
Glyma17g32610	MYB/HD-like
Glyma08g38490	ZF-HD

List of transcription factors identified to be unique to *G. max *and deleted in *G. soja*.

### Unique to *G. soja*

The sequences of 22,927 new contigs unique to *G. soja *(not present in *G. max*) were compared against the entire set of CDS sequences for *Arabidopsis thaliana *from TAIR10 [16], as well as Arabidopsis gene lists associated with seed, fatty acid metabolism, and protein metabolism and biosynthesis for sequence similarity. We used a filtering criteria for this comparison using Blast where hits passing the threshold of identity > = 80%, alignment length > = 100 bps, and e-value < 1e-3 were considered as significant hits. We identified 148, 132, 6, and 2 unique contigs matching significant hits from CDS, related to seed, fatty acid metabolism, and protein metabolism and biosynthesis, respectively. This was used for identifying the associated annotations and we also identified the pathways they were associated with.

### SNPs analysis

The total number of all SNPs in *G. soja *identified from the MAQ analysis is 2,504,985 [1]. The number of non-synonymous SNPs amongst them is 56,948 and synonymous SNPs account for remaining 2,448,037. Out of these 23,379 non-synonymous SNPs in *G. soja *correspond to 10,770 unique genes annotated with gene IDs ending in ".1" and have one or multiple amino acid substitutions. Polyphen (Polymorphism Phenotyping) [17] and SIFT (Sorting Intolerant from Tolerant) [18] analysis was conducted for these data, to identify the significant non-synonymous SNPs affecting the protein characteristics. SNP positions with normalized probabilities less than 0.05 are predicted to be deleterious, those greater than or equal to 0.05 are predicted to be tolerated by the SIFT analysis.

We considered only the 66,365 Glyma1 gene model predictions with IDs ending in ".1" for further analysis. We compared the positions of these SNPs against the gene model positions and looked for SNPs overlapping within 1,000 bp upstream regions of these genes. Out of these SNPs, 174,208 SNPs were identified to be in regions 1 kb upstream of 46,270 genes (mean: 3.77 SNPs per kb, SD: 3.22). Out of these, 1,177 appear in 279 soybean genes (mean: 4.22, SD: 3.53) that are missing in *G. soja *but present in *G. max*, and remaining 173,031 SNPs overlap with 45,991 remaining common genes (mean: 3.77, SD: 3.21). The details are shown in Table 2 below. We further applied a stringent filtering criterion of number of SNPs in 1 kb upstream region > = 12 (p = 0.00467), to select these genes for further analysis. 14 genes (as listed below in Table 3) from 425 genes unique to *G. max *and 1533 genes from the remaining 45,991 common genes met the criterion and the annotations were studied for each of these genes.

**Table 2:** Distribution of SNPs positions amongst genes unique to *G. max *and common to both.

Region (# of genes)	# of SNPs (# of genes)	Average	Standard deviation
425 deleted genes (425)	1177 (279)	4.22	3.53
Remaining genes (65,940)	173,031 (45,991)	3.76	3.21
Total (66,365)	174,208 (46,270)	3.77	3.22

Details of average and standard deviation of SNP distribution for genes, unique to *G. max *and common to both varieties of soybean.

**Table 3:** 14 genes unique to *G. max *and having > = 12 SNPs in 1 kb upstream region.

Genes	Functional Annotations
Glyma01g04240.1	PF00931|PF00560|PF00560|PF00560|PF00560|PF00560|PF00560 NB-ARC domain|Leucine Rich Repeat|Leucine Rich Repeat|Leucine Rich Repeat|Leucine Rich Repeat|Leucine Rich Repeat|Leucine Rich Repeat
Glyma03g05320.1	
Glyma05g21440.1	PF00069|PF07714 Protein kinase domain|Protein tyrosine kinase
Glyma09g29090.1	
Glyma12g09780.1	PF00106 short chain dehydrogenase
Glyma16g06560.1	
Glyma16g24930.1	
Glyma17g29450.1	PF01885 RNA 2'-phosphotransferase, Tpt1/KptA family
Glyma17g33900.1	PF00400|PF00400|PF00400|PF00400 WD domain, G-beta repeat|WD domain, G-beta repeat|WD domain, G-beta repeat|WD domain, G-beta repeat
Glyma18g42150.1	
Glyma18g48950.1	PF00560|PF00560|PF00560|PF00560|PF00560|PF00560|PF00560|PF00560|PF00560|PF00560|PF00560|PF00069|PF07714 Leucine Rich Repeat|Leucine Rich Repeat|Leucine R ich Repeat|Leucine Rich Repeat|Leucine Rich Repeat|Leucine Rich Repeat|Leucine Rich Repeat|Leucine Rich Repeat|Leucine Rich Repeat|Leucine Rich Repeat|Leucine Rich Repea t|Protein kinase domain|Protein tyrosine kinase
Glyma18g51960.1	PF00931 NB-ARC domain
Glyma19g07650.1	PF01582|PF00931|PF05729|PF00560|PF00560|PF00560|PF00560|PF00560|PF00560|PF00560|PF00560|PF00560 TIR domain|NB-ARC domain|NACHT domain|Leucine Rich Repeat |Leucine Rich Repeat|Leucine Rich Repeat|Leucine Rich Repeat|Leucine Rich Repeat|Leucine Rich Repeat|Leucine Rich Repeat|Leucine Rich Repeat|Leucine Rich Repeat
Glyma20g02020.1	PF00795 Carbon-nitrogen hydrolase

List of genes unique to *G. max *with > = 12 SNPs in 1 kb upstream region.

### Non-synonymous SNPs (in exons, upstream regions) analysis

The SNPs distribution from Kim *et al.*[1] was utilized in further analysis. The number of SNPs identified in exon regions is 86,236 and those identified in non-exon regions is 251,021. These were identified to be in 36,861 and 40,282 genes respectively. We calculated the difference between the SNP distribution observed in the 1 kb exon and non-exon regions to look for any distribution biases. We defined,

$$\begin{array}{l} \text{Average for 1 kb exon region = (\# of SNP in exon}/\text{length of exon)*1000}\\ \text{Average for 1 kb non}-\text{exon region = (\# of SNP in non}-\text{exon}/\text{length of non}-\text{exon)*1000} \end{array}$$

Thus,

Difference in SNPs in 1 kb region = Average for 1 kb exon region- Average for 1 kb non-exon region.

2,405 genes met the filtering criteria with difference in SNPs in 1 kb region > 0 and # of SNPs in 1 kb exon regions > 10 (1% alteration). These 2,405 genes were further compared against the non-synonymous SNPs list to study and identify genes with higher # of non-synonymous SNPs. The ratio of # of non-synonymous over synonymous SNPs was calculated for this analysis.

Ratio = (# of non-synonymous SNPs / # of synonymous SNPs)

139 genes were identified that met the criteria of Ratio > 1. The pathways where these 139 genes were involved in were identified by using Gene Pathway Viewer in SoyKB [9]. Fatty acid biosynthesis, amino acid metabolism and biosynthesis, and protein trafficking and translation pathways are the major pathways these genes fall under.

### Upstream region analysis

The 1 kb upstream sequence of 60 genes out of 425 unique genes in *G. max *with at least one non-synonymous deleterious SNPs and 139 genes with difference> 0, average> 10, and ratio of # of non-synonymous SNPs and # of synonymous SNPs> 1 were extracted and were further analysed using the PLACE database [19] to look for any cis-elements sequences in these regions. The numbers of SNPs in 1 kb upstream for each gene causing changes in the cis-elements were further studied and the detailed information of position and sequence of affected cis-elements was generated.

### Homeologous genes analysis

9,807 duplicated homolog pairs from Libault et al. [20] were compared to 425 unique genes and 139 genes with non-synonymous SNPs in *G. max *as described above. 32 duplicated homolog pairs were identified with one homolog deleted and 18 duplicated homolog pairs were identified with one homolog with non-synonymous/synonymous SNPs ratio > 1. Table 4 and Table 5 show the list of these genes respectively. The functional annotations for all these genes were acquired using multiple gene search tool in SoyKB. Genes associated with storage protein biosynthesis and heat shock protein related chaperon classes were mostly deleted among these 32 homolog pairs. This result indicates that the wild soybean genome has retained a good portion of unique regions, when compared to *G. max*, and this suggests that *G. max *genome has undergone deletions during the domestication process.

**Table 4:** List of 32 genes in *G. max *with one homolog deleted.

Homolog 1	Homolog 2	Functional Annotation
Glyma03g04510	Glyma01g32400	Serine/threonine protein kinase
Glyma06g40740	Glyma06g40710	Apoptotic ATPase
Glyma07g07920	Glyma03g01500	ATP-dependent RNA helicase
Glyma07g07930	Glyma03g01510	Mitochondrial ribosomal protein S27
Glyma07g33040	Glyma02g15450	PX domain|Sorting nexin C terminal
Glyma07g33050	Glyma02g15430	
Glyma08g23120	Glyma07g02990	Flavonol reductase/cinnamoyl-CoA reductase
Glyma11g08350	Glyma01g36890	3-dehydroquinate synthase
Glyma12g10680	Glyma06g46040	Plant protein family
Glyma13g20100	Glyma10g05750	Dolichyl pyrophosphate phosphatase and related acid phosphatases
Glyma13g24010	Glyma07g32580	RIBOSOMAL RNA PROCESSING PROTEIN 7-RELATED
Glyma13g36030	Glyma12g34480	GH3 auxin-responsive promoter
Glyma13g37110	Glyma12g33320	Zinc finger C-x8-C-x5-C-x3-H type (and similar)|Zinc finger C-x8-C-x5-C-x3-H type (and similar)
Glyma14g02210	Glyma02g46440	CDP-diacylglycerol synthase
Glyma15g23400	Glyma09g11600	PAS fold|GAF domain|Phytochrome region|PAS fold|PAS fold|PAS fold|His Kinase A (phosphoacceptor) domain |Histidine kinase-, DNA gyrase B-, and HSP90-like ATPase
Glyma16g01990	Glyma07g05420	Iron/ascorbate family oxidoreductases
Glyma16g32760	Glyma09g27910	40S ribosomal protein S14
Glyma16g33630	Glyma09g29090	Uncharacterized conserved protein
Glyma17g11920	Glyma13g22960	Endonuclease/Exonuclease/phosphatase family|Reverse transcriptase (RNA-dependent DNA polymerase)
Glyma17g17000	Glyma05g23000	Methyltransferase domain
Glyma17g18180	Glyma05g21440	Serine/threonine protein kinase
Glyma17g33900	Glyma14g11930	Histone H3 (Lys4) methyltransferase complex and RNA cleavage factor II complex, subunit SWD2
Glyma18g16370	Glyma08g40730	H+/oligopeptide symporter
Glyma18g29500	Glyma08g38260	
Glyma18g45060	Glyma09g40750	CYTOCHROME P450
Glyma18g45070	Glyma09g40750	CYTOCHROME P450
Glyma19g00280	Glyma05g08780	Retrotransposon gag protein|Reverse transcriptase (RNA-dependent DNA polymerase)|Integrase core domain|'chromo' (Chromatin Organisation Modifier) domain|S1 RNA binding domain|S1 RNA binding domain
Glyma19g25070	Glyma16g06460	UPF0041 BRAIN PROTEIN 44-RELATED
Glyma19g37230	Glyma03g34540	Uncharacterized conserved protein
Glyma20g01800	Glyma07g34250	Cytochrome P450 CYP2 subfamily
Glyma20g02020	Glyma06g05770	Carbon-nitrogen hydrolase
Glyma20g23760	Glyma10g43130	Thioredoxin-like protein

List of 32 gene pairs where one homolog is deleted in *G. soja*.

**Table 5:** List of 18 genes in *G. max *with one homolog with non-synonymous/synonymous SNPs ratio > 1.

Homolog 1	Homolog 2	Functional Annotation
Glyma03g04950	Glyma01g32160	Protease inhibitor/seed storage/LTP family
Glyma06g14000	Glyma04g40790	Molecular chaperone (small heat-shock protein Hsp26/Hsp42)
Glyma06g19010	Glyma04g35940	Uncharacterized conserved protein, contains JmjC domain
Glyma06g40780	Glyma06g40740	Apoptotic ATPase
Glyma07g15110	Glyma01g00900	HAD superfamily, subfamily IIIB (Acid phosphatase)
Glyma08g14990	Glyma05g31750	PPR repeat|PPR repeat|PPR repeat|PPR repeat|PPR repeat|PPR repeat|PPR repeat
Glyma09g32240	Glyma07g09560	Uncharacterized conserved protein
Glyma11g09160	Glyma01g36280	Histidine kinase-, DNA gyrase B-, and HSP90-like ATPase
Glyma12g07280	Glyma11g20640	SUA5
Glyma15g09420	Glyma13g29590	Molecular chaperones GRP78/BiP/KAR2, HSP70 superfamily
Glyma15g10430	Glyma13g28680	gb def: putative formate dehydrogenase alpha subunit [thermococcus litoralis]
Glyma15g20000	Glyma09g08370	ATP-dependent RNA helicase
Glyma16g02950	Glyma07g06310	HISTONE H3
Glyma18g40190	Glyma07g16190	Iron/ascorbate family oxidoreductases
Glyma18g40210	Glyma07g16190	Iron/ascorbate family oxidoreductases
Glyma19g31230	Glyma03g28480	Pyrroline-5-carboxylate reductase
Glyma19g33520	Glyma03g30620	PNAS-3 RELATED
Glyma20g22690	Glyma10g28600	Predicted ABC-type transport, ATPase component/CCR4 associated factor

List of 18 gene pairs in *G. max*, with ratio of non-synonymous/synonymous SNPs > 1 in one homolog.

### Genes involved in seed development, oil, protein and other functional groups of interest

**Figure 1:**
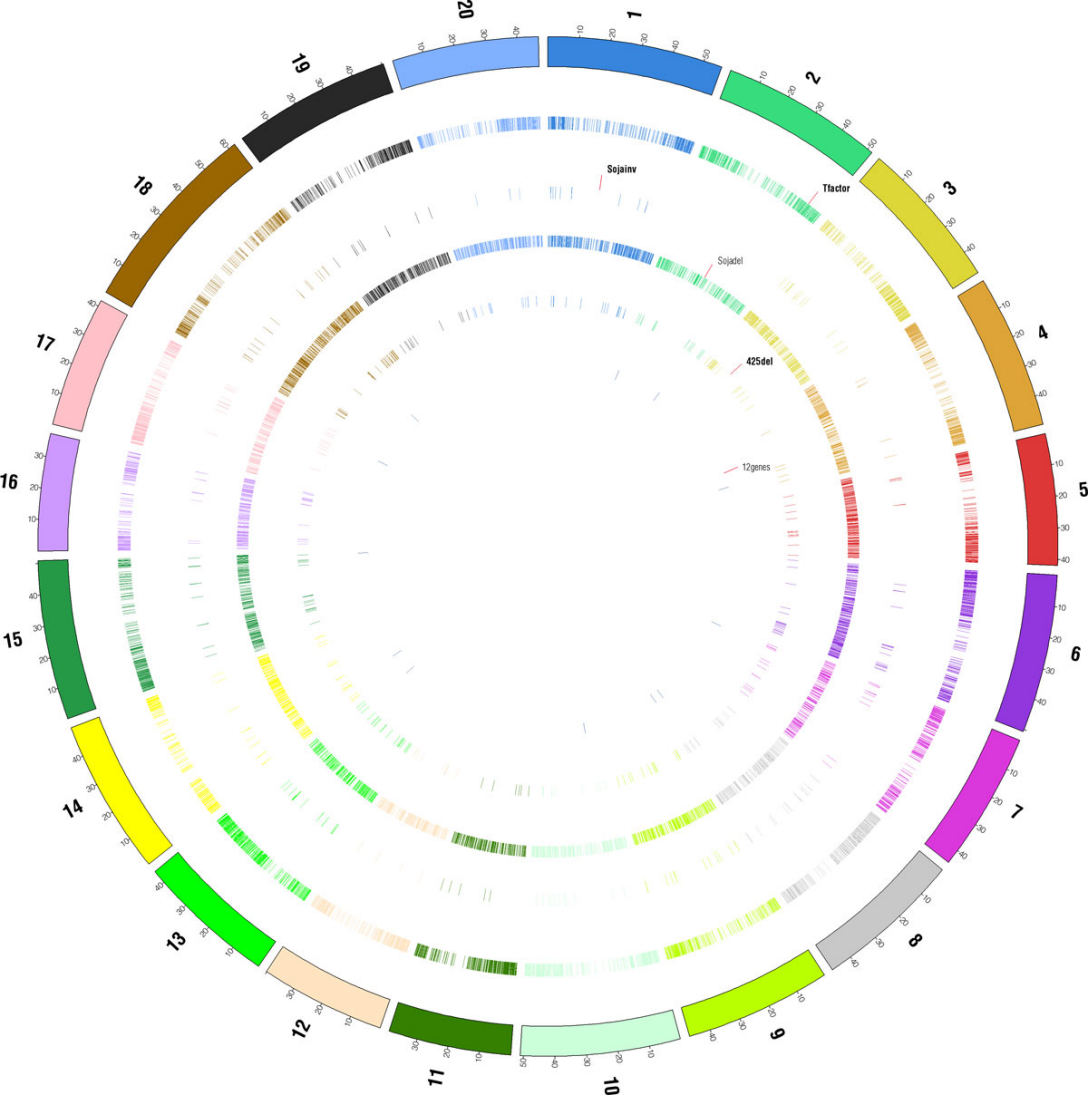
**Circular genome viewer created using Circos**. Circular genome viewer created using Circos, showing of inversions, deletions, unique genes and transcription factors in *G. max*, along with the QTL regions for various traits. The QTLs for oil trait is shown in orange, protein trait in red and seed trait in pink.

We analyzed list of genes for various functions of our interest including seed development, oil-related, protein-related and some other functional groups of interest. The analysis was conducted to identify any unique genes in *G. max *involved in these functions that may have been deleted in *G. soja*. Figure 1 shows the positions of inversions, deletions, unique genes and TFs in *G. max*, along with the QTL regions for various traits in a circular genome viewer using Circos [21] tool.

### Seed development

A list of 16,365 unique loci involved in seed in *Arabidopsis thaliana *was extracted based on keyword search at TAIR [16]. These sequences were compared against the soybean cDNA sequences to identify the corresponding homolog using Blast (criteria: identity > = 80%, aligned length > = 100 bps, and e-value < 1e-3). When compared against the 425 soybean genes that are deleted in *Glycine soja*, 12 genes involved in seed development that are unique to *G. max *were identified as listed in Table 6.

**Table 6:** List of 12 genes involved in seed development that are unique to *G. max*.

Genes	Associated traits
Glyma03g34540.1	Salt tolerance
Glyma04g12020.1	Protein
Glyma08g22840.1	Fructose content, Sclerotinia
Glyma09g15890.1	Arabinose, Sclerotinia, Seed composition, Seed weight, Yield
Glyma09g16680.1	Arabinose, Sclerotinia, Seed composition, Seed weight, Yield
Glyma09g30700.1	Arabinose, Palmitic
Glyma10g31080.1	Seed weight, Peanut root-knot nematode resistance
Glyma13g17820.1	Seed weight, Peanut root-knot nematode resistance
Glyma14g02210.1	
Glyma14g10940.1	Soybean cyst nematode resistance
Glyma14g33630.1	Iron efficiency, Seed nitrogen
Glyma18g07560.1	Seed weight, Soybean cyst nematode resistance

List of 12 genes unique to *G. max *that are involved in seed development functions.

Soybean gene Glyma04g12020.1 is annotated as WD40 repeat proteins and these proteins regulate biosynthesis of anthocyanins, proanthocyanidins (PAs), and mucilage in the seed and seed developmental stages [22]. Functional classification of these 12 genes clearly shows their roles in major biochemical processes during seed development in plants. Various molecular functions of these genes such as sugar and carbohydrate metabolism, nitrogen assimilation and metabolism, transporters, trans-membrane, and even the regulation of ubiquitination of proteins [23] are essential to regulate seed developmental processes and seed size in soybean. Above all, the Glyma14g02210.1 encoding enzyme CDP-diacylglycerol synthase is a major enzyme in regulation of fatty acyl fluxes during triacylglycerol (TAG) biosynthesis [24].

### Oil-related

The sequences of 694 unique loci of known or genes considered to be involved in acyl lipid metabolism of *Arabidopsis thaliana *were compared against the soybean cDNA sequences to identify the corresponding homolog using Blast (criteria: identity > = 80% and e-value < 1e-3). When compared against the 425 soybean genes that are deleted in *G. soja *but present in *G. max*, 2 genes involved in acyl lipid metabolism were identified that are unique to *G. max*. 1,261 genes out of 1,266 unique hits to genes involved in acyl lipid metabolism were mapped to genes with ".1" gene models. As shown in Table 7, 3 genes were found in the list of 425 unique genes in *G. max *and one gene (Glyma10g05750.1) is novel when compared to the previous analysis. The gene Glyma06g39750.1 is shown to overlap with oil, seed-weight and yield related traits QTLs. The gene Glyma10g05750.1 is associated with southern root-knot nematode resistance trait and falls in its QTL. Glyma10g05750.1 is dolichyldiphosphatase which is catalytic and membrane related. This enzyme is reported as cell cycle dependent in human and, dolichol and dolichyl phosphate phosphatase (polyphenols) were characterized from soybean previously [25]. The lipid fractions of the lipo-sugar carriers are essential for the biosynthesis of N-linked glycoproteins. The gene Glyma06g39750.1 encodes for 3-hydroxyacyl-coa dehydrogenase and this enzyme catalyzes the third reaction of the mitochondrial β-oxidation cascade in animals. The gene Glyma04g12260 encodes for the fatty acyl-coenzyme A reductases. Earlier studies has confirmed that the heterologous expression of fatty acyl-coenzyme A reductases FAR1, FAR4, and FAR5 in yeast are active alcohol-forming FARs with distinct, but overlapping, chain length specificities ranging from C18:0 to C24:0 [26]. This report indicates that Arabidopsis FAR1, FAR4, and FAR5 generate the fatty alcohols found in root, seed coat, and wound-induced leaf tissue. The soybean transcript for Phospholipase D (Glyma07g08740) belongs to a lipolytic enzyme subclass which catalyzes the hydrolysis and transesterification of glycerophospholipids at the terminal phosphodiester bond [27].

**Table 7:** List of genes involved in acyl lipid metabolism that are unique to *G. max*.

Genes	Associated Traits
Glyma06g39750.1	Oil, Seed weight, Yield
Glyma14g02210.1	
Glyma10g05750.1	Southern root-knot nematode resistance

List of 3 genes unique to G. max involved in acyl lipid metabolism.

### Protein-related

Out of the 425 unique gens is *G. max*, we have identified several candidates associated with amino acid biosynthesis, amino acid transporters, nitrogen metabolism and glutamate biosynthesis directly correlated with the protein biosynthesis in soybean. One of the major groups of genes belongs to the cellular amino acid biosynthetic processes, which are the basic building blocks and very crucial for protein biosynthesis and metabolism in plants. Some example are, Glyma19g16450 encoding for glutamate synthase, Glyma08g07140 encoding for glutamine amidotransferase class-I, Glyma10g05090 ArgJ family proteins (glutamate ornithine acetyl transaminases). Another group of transcripts predominantly related to the protein metabolism are the vacuolar transporting proteins. Zouher et al. [28] have shown that VSR1, VSR3 and VSR4, but not the remaining VSRs or RMRs, participate in vacuolar sorting of VAC2 in vegetative tissues, and 12S globulins and 2S albumins in seeds, an activity that is essential for seedling germination vigor. Genes encoding for ribosomal protein, eg. Glyma19g06460, are another abundant group came out of the analysis. Ribosomes are the molecular machine for protein biosynthesis and the large (50S) and small (30S) ribosomal subunits contain over 50 distinct ribosomal (r) proteins that interact with the rRNAs and with one another [29]. Transcript Glyma09g36700 encodes for the signal recognition factor. Zhang and Shan [30] reported that the signal recognition particle (SRP) is universally conserved cellular machinery responsible for delivering membrane and secretory proteins to the proper cellular destination. The precise mechanism by which fidelity is achieved by the SRP pathway within the in vivo environment is yet to be understood.

### Others functional groups of interest

360 unique loci involved in ubiquitin-dependent catabolic process, 5 unique loci involved in SCF-dependent proteasomal ubiquitin-dependent protein catabolic process, 39 unique loci involved in regulation of translation, 1 unique locus involved in regulation of protein catabolic process, 4 unique loci involved in positive regulation of translation, 8 unique loci involved in negative regulation of translation, 79 unique loci involved in glycoprotein biosynthetic process, and 247 unique loci involved in cellular amino acid biosynthetic process were also mapped to soybean gene models using Blast with best hits with e-value < 1e-3 and 292, 4, 36, 1, 4, 7, 75, and 232 soybean genes were identified, respectively. When compared against the 425 soybean genes that are deleted in *G. soja *but present in *G. max*, no genes involved in protein metabolism and regulation were identified. When compared against the 139 significant non-synonymous genes, one gene (Glyma03g28480.1) involved in cellular amino acid biosynthetic process was identified.

### QTL, traits and SNPs analysis

The list of non-synonymous SNPs analyzed by Polyphen and Sift was further studied and compared against the different QTLs for various traits such as seed, oil, protein, etc. The genes falling in these QTLs of interest were studied in further details for the difference between the *G. max *and *G. soja*. The QTL regions for all the three traits were acquired using Breeding Program and Chromosome Visualizer tools of SoyKB [9]. The total # of all SNPs in *G. soja *is 2,504,985. Out of these 594,328 overlap with seed trait QTLs; 316,447 overlap with oil trait QTLs; and 380,719 overlap with protein trait QTLs. Each of these traits and QTLs were further analyzed for identifying overlapping genes unique to *G. max *and deleted in *G. soja*.

When further analyzed, we identified 315 out of the 425 unique genes in *G. max *also overlapping with various QTL regions for different traits. Out of these, we identified 97 genes overlapping with the seed trait QTLs, 54 with the Oil trait QTLs and 65 with the Protein trait QTLs in *G. max*.

### Non-synonymous SNPs overlapping QTLs

Analysis was further conducted to identify the overlap on the non-synonymous SNPs with the QTL regions of various traits. The 23,379 non-synonymous SNPs in *G. soja *corresponding to 10,770 unique genes were utilized for this analysis and compared against various traits and QTL regions. When compared against the unique list of 20,498 genes ending in ".1" and falling in various QTLs for all available traits, we identified non-synonymous SNPs in 20,031 unique genes. Non-synonymous SNPs are present in 3,221, 1,579 and 2,346 genes falling in the QTLs of seed, oil and protein traits, respectively.

### SoyKB graphical chromosome visualizer

**Figure 2:**
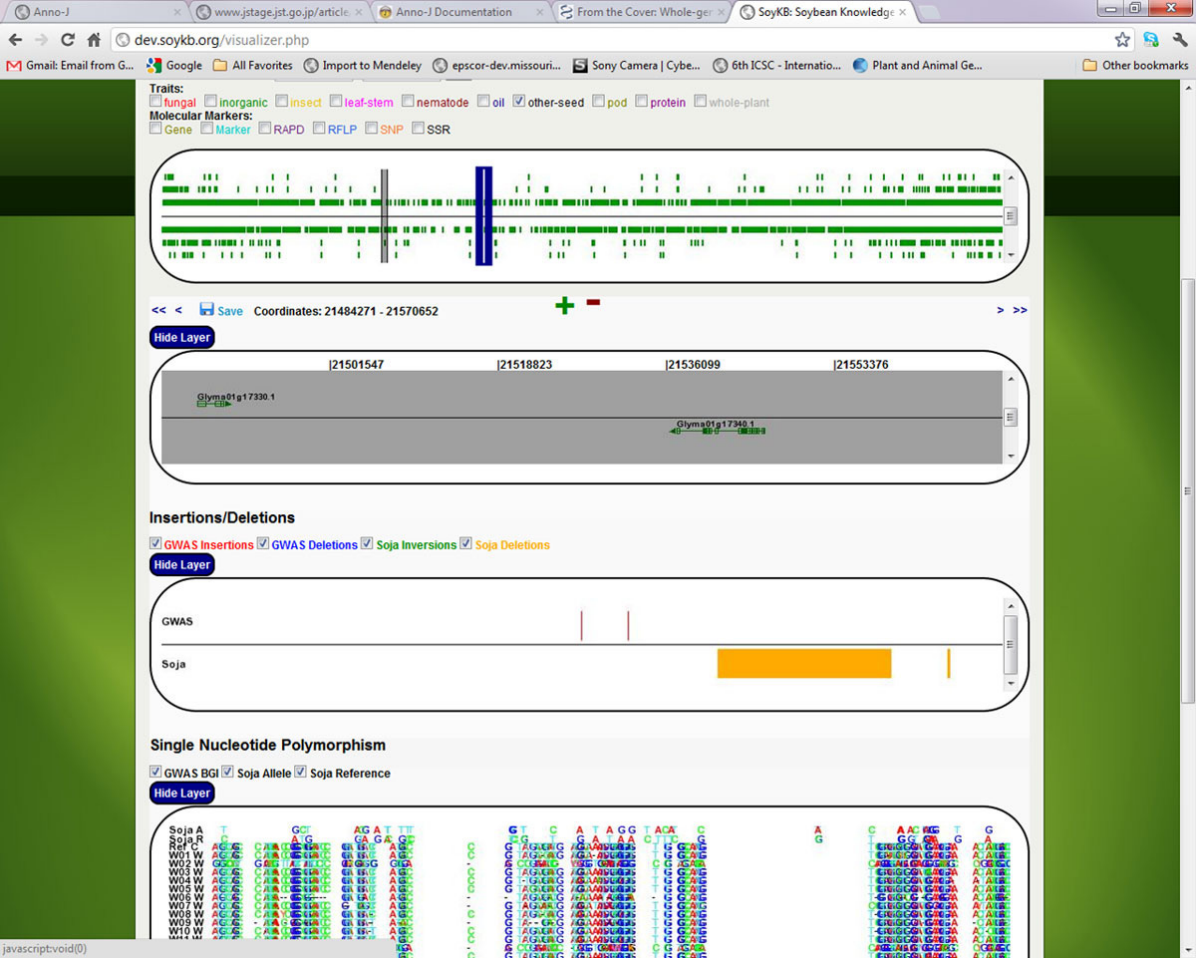
**Chromosome visualizer tool in SoyKB showing the gene Glyma01g17340**. Chromosome visualizer tool in SoyKB shows the gene Glyma01g17340.1, which is unique in G. *max*, overlapping in QTL region for seed trait as well as the deletion region in *G. soja*.

We performed some analysis and visualization of the QTLs and traits using the SoyKB web resource. The *G. soja *SNPs, inversions and deletions have been added to the SoyKB resource. The graphical chromosome visualizer is an innovative molecular breeding tool, developed as part of SoyKB Breeding Program Tools, which integrates the genomic variations data such as SNPs and GWAS alongside the traits, QTLs, molecular markers and underlying genes. It displays the data linearly for each chromosome along with other useful information such as QTLs for multiple traits, SNPs from multiple genotypes of soybean including *G. soja*, as well as insertion, inversion and deletions data from multiple genotypes and *G. soja*. The chromosome visualizer can be directly accessed via the Tools menu on SoyKB (http://soykb.org/visualizer.php). This tool can be used for highly meaningful analysis for QTLs of specific traits and showing the overlapping genes and regions of deletion and SNPs in comparison to the Williams82 reference genome. The list of genes falling in any QTL regions for single or multiple traits can be saved using the "Save" option on the viewing panel or "Left-clicking" the highlighted QTL region. The panels can be dragged and dropped to rearrange the sequence of visualizing the data. We performed the combined analysis for all the 3 traits. For example, in seed trait QTLs, gene Glyma01g17340.1 is deleted in *G. soja *and thus unique to *G. max *as shown in Figure 2. There are 315 such other genes falling in QTLs and being unique to *G. max*.

### Multiple traits and QTL analysis

**Figure 3:**
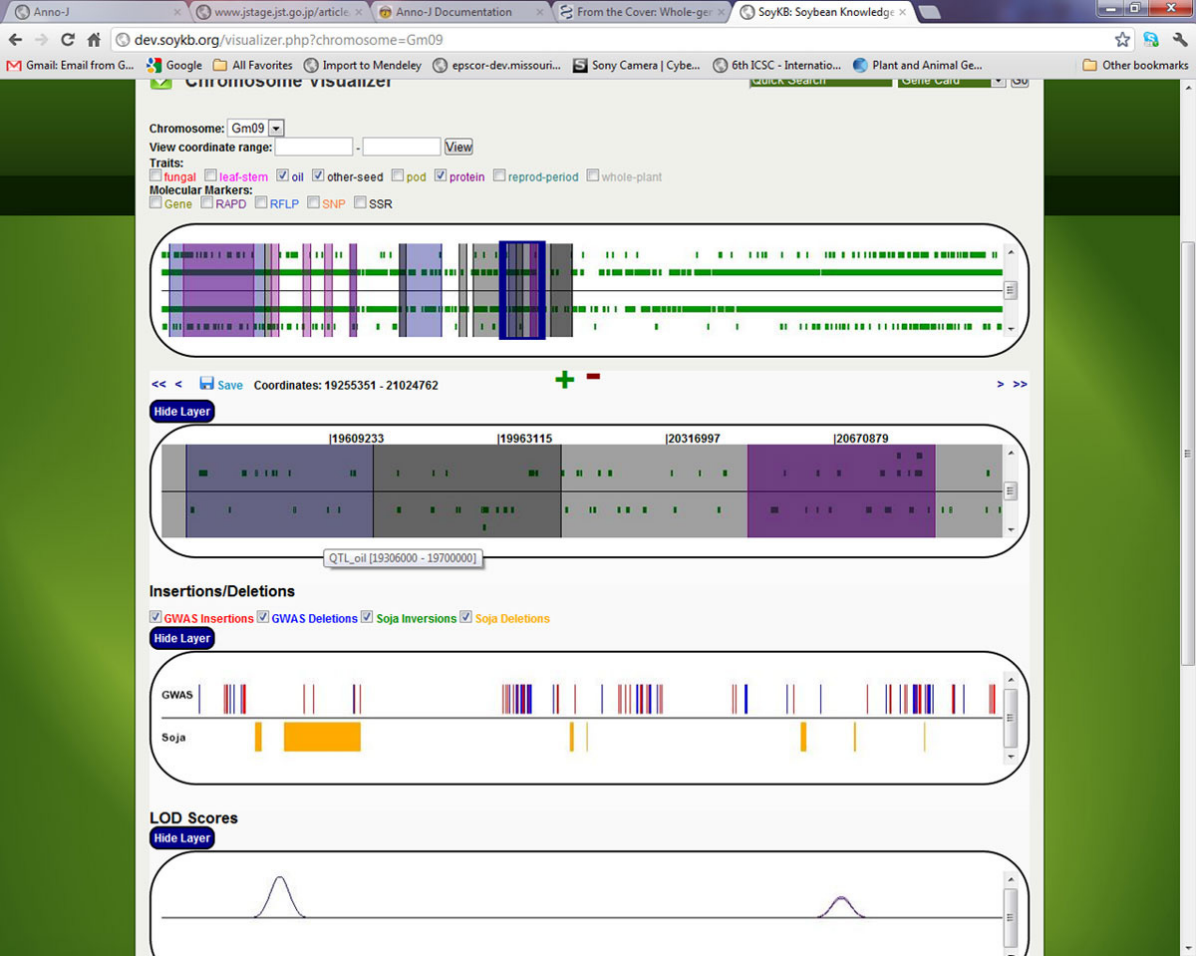
**Chromosome visualizer tool in SoyKB showing the region of chromosome Gm09**. Chromosome visualizer tool in SoyKB shows the region of chromosome Gm09 with overlapping QTL regions for multiple traits. Oil trait is shown in pink, protein trait in blue and seed trait in grey.

We selected the seed, oil and protein traits to visualize the QTLs for all three of them. We also selected the *G. soja *SNPs as well as inversion and deletions datasets from the bottom panels for viewing. Figure 3 shows the region of Gm09 chromosome with the overlapping QTLs for all the 3 traits and also the region overlapping with the *G. soja *deletions region. This clearly shows an example of genes unique to *G. max *and to be involved important functions related to seed, oil and protein traits. This tool can be used by researchers to identify, confirm as well as generate lists of genes falling in the QTL regions.

Combined analysis of seed and oil traits shows a region of deletion in *G. soja *on chromosome Gm01:19,434,434 - 19,498,249, which overlaps with the QTL regions for both the traits. This involves 7 consecutive genes (Glyma09g16400, Glyma09g16410, Glyma09g16420, Glyma09g16430, Glyma09g16440, Glyma09g16470, and Glyma09g16480), which are deleted in *G. soja *and are significant in *G. max *as they all fall in the QTL region for both oil and seed traits (Figure 3), contributing to the differences in the oil and seed development between the two varieties. Combined analysis of oil and protein traits shows a QTL region on chromosome Gm09 that contains several genes deleted in *G. soja*. Several genes in this region are significant since they overlap with some deleted regions in *G. soja *and are unique to *G. max*, in addition to falling in important QTL regions. In Figure 3, the oil trait QTL is highlighted in pink and protein QTL in blue. The functional annotation and experimental data for all of these genes were acquired using the multiple gene search feature of SoyKB.

## Discussion/conclusions

With the availability of genome sequence data for both the varieties, it added tremendous valuable data that can be mined in an integrative and innovative manner to draw significant and interesting conclusions that can directly impact breeding of better soybeans. Some analysis has been done on the differences between *G. soja *and *G. max *earlier in terms of flowering, carbon metabolism and disease resistance [31]. However, no comparisons have been done in terms of differences in oil, protein and seed related functions and traits, especially at the genomic level. We have mined this data comprehensively for the traits of interest especially oil, seed development and protein. Our analysis shows there are some significant differences between *Glycine max *and *Glycine soja *at the genomic level, which corresponds with some functionally important genes for seed, oil and protein traits. These genes can explain some of the phenotypic differences observed between *G. soja *and *G. max *especially in terms of the above three traits. More experimental validations will follow including knockout studies of these genes in *G. max *to see the associated changes in phenotype. It is understood that the genomic differences identified in this study were revealed by comparing genome sequence of single genotype of *G. soja *and *G. max*. There are variations that exist within different populations of *G. soja *and *G. max*, respectively. Further studies comparing more diverse populations of both *G. soja *and *G. max *will be needed and will be extremely valuable in confirming some of these differences identified from a single population study. Nevertheless, the methodology demonstrates strongly how the genomic sequencing data can be combined with QTL and trait information to further narrow unique gene candidates involved in specific functional processes.

As part of the analysis more novel and innovative tools were developed as part of Breeding Program suite of tools in SoyKB, which are beneficial towards analysing and visualizing various traits and genes overlapping with them. This is a resource which is available to all communities freely and so our development can be utilized by others in the soybean area including researchers, scientists, breeders and farmers to their advantage. The overlapping of other genomic variations data along with the above information makes drawing inferences very easy and makes it the most informative tool available thus far.

## Competing interests

The authors declare that they have no competing interests.

## Authors' contributions

TJ conducted the bioinformatics analysis and downstream data analysis for QTL and traits. TJ is also the lead developer for the SoyKB web resource and drafted the initial manuscript. BV provided the expert advice for selection of traits and functional categories to study. Both TJ and BV contributed equally to the work. JW performed the sequence similarity analysis and SNPs comparison analysis. SL collaborated with the team and provided the sequences and associated files for *G. soja*. HTN and DX provided guidance and valuable insight. All authors have read and approved the manuscript.

## Declarations

The funding for the publication of this article came from United Soybean Board (project 8236).

This article has been published as part of *BMC Genomics *Volume 14 Supplement 1, 2013: Selected articles from the Eleventh Asia Pacific Bioinformatics Conference (APBC 2013): Genomics. The full contents of the supplement are available online at http://www.biomedcentral.com/bmcgenomics/supplements/14/S1.

## Supplementary Material

Additional file 1 **Supp Table 1**. List of 425 genes found to be unique to *G. max *when compared to *G. soja*. Click here for file
